# The long-term impact of folic acid in pregnancy on offspring DNA methylation: follow-up of the Aberdeen Folic Acid Supplementation Trial (AFAST)

**DOI:** 10.1093/ije/dyy032

**Published:** 2018-03-12

**Authors:** Rebecca C Richmond, Gemma C Sharp, Georgia Herbert, Charlotte Atkinson, Caroline Taylor, Sohinee Bhattacharya, Doris Campbell, Marion Hall, Nabila Kazmi, Tom Gaunt, Wendy McArdle, Susan Ring, George Davey Smith, Andy Ness, Caroline L Relton

**Affiliations:** 1 MRC Integrative Epidemiology Unit, University of Bristol, Bristol, UK; 2 Population Health Sciences, Bristol Medical School, University of Bristol, Bristol, UK; 3 NIHR Biomedical Research Centre (Nutrition Theme), University of Bristol, Bristol, UK; 4 Bristol Dental School, University of Bristol, Bristol, UK; 5 Centre for Child and Adolescent Health, University of Bristol, Bristol, UK; 6 Institute of Applied Health Sciences, University of Aberdeen, Aberdeen, UK

**Keywords:** epigenetic, AFAST, randomized–controlled trial, longitudinal, epigenome-wide association study, DNA methylation

## Abstract

**Background:**

It has been proposed that maternal folic-acid supplement use may alter the DNA-methylation patterns of the offspring during the *in-utero* period, which could influence development and later-life health outcomes. Evidence from human studies suggests a role for prenatal folate levels in influencing DNA methylation in early life, but this has not been extended to consider persistent effects into adulthood.

**Methods:**

To better elucidate the long-term impact of maternal folic acid in pregnancy on DNA methylation in offspring, we carried out an epigenome-wide association study (EWAS) nested within the Aberdeen Folic Acid Supplementation Trial (AFAST—a trial of two different doses: 0.2 and 5 mg, folic acid vs placebo). Offspring of the AFAST participants were recruited at a mean age of 47 years and saliva samples were profiled on the Illumina Infinium Human Methylation450 array. Both single-site and differentially methylated region analyses were performed.

**Results:**

We found an association at cg09112514 (*p* = 4.03×10^–9^), a CpG located in the 5’ untranslated region of *PDGFRA*, in the main analysis comparing the intervention arms [low- (0.2 mg) and high-dose (5 mg) folic acid combined (*N* = 43)] vs placebo (*N* = 43). Furthermore, a dose–response reduction in methylation at this site was identified in relation to the intervention. In the regional approach, we identified 46 regions of the genome that were differentially methylated in response to the intervention (Sidak *p-value* <0.05), including *HLA-DPB2*, *HLA-DPB1*, *PAX8* and *VTRNA2–1*. Whereas cg09112514 did not replicate in an independent EWAS of maternal plasma folate, there was suggested replication of differential methylation in *PAX8*.

**Conclusions:**

The results of this study suggest that maternal folic-acid supplement use is associated with changes in the DNA methylation of the offspring that persist for many years after exposure *in utero*. These methylation changes are located in genes implicated in embryonic development, immune response and cellular proliferation. Further work to investigate whether these epigenetic changes translate into detectable phenotypic differences is required.

**Key Messages**

- We investigated the impact of a folic-acid supplementation trial that enrolled pregnant women in the late 1960s on long-term epigenetic changes in their offspring by assessing differences in DNA-methylation levels of their offspring at a mean age of 47 years.
- In saliva samples obtained from the offspring 47 years after the trial was conducted, we identified 45 regions of the genome that were differentially methylated in response to the intervention.
- The results of the study suggest that maternal folic-acid supplement use is associated with changes in DNA methylation that persist for many years after *in-utero* exposure, but further work is needed to investigate whether these epigenetic changes translate into detectable phenotypic differences.

## Introduction

Folate is an essential micronutrient that plays an important role in fetal development,^1^ with the potential for lifelong consequences.^2^ It is a key player in one-carbon metabolism that is closely linked to the provision of methyl groups for the methylation of DNA^3^—an epigenetic process that is crucial in early development.^4^ Therefore, it has been proposed that maternal folic acid may alter the methylation patterns of the offspring during the *in-utero* period, which could impact health outcomes in later life. This was demonstrated in the *Agouti* mouse model, where methyl donor supplements (including folate) given to pregnant dams resulted in increased DNA methylation in the offspring at the *Agouti* allele, which had phenotypic consequences of shifting offspring coat colour and reducing the risk of obesity and tumorigenesis.^5^

Evidence from human studies suggests a role for prenatal folate levels in influencing DNA methylation in neonates and children,^6–9^ but this has not been extended to consider the persistent effects of such exposures into adulthood. To examine effects in adults, studies with long-term follow-up are required. Furthermore, observational studies investigating the impact of nutritional exposures on the epigenome are often confounded, e.g. by other highly correlated macro/micronutrients or socio-economic factors not adequately captured in these previous studies. The strongest evidence relating prenatal nutrition to offspring methylation derives from intervention studies (randomized–controlled trials or natural experiments). In these studies, large differences in nutritional status occur in the study population (largely) at random and are therefore unlikely to be associated with confounding factors.^7^^,^^10^^,^^11^

To better elucidate the long-term impact of maternal folic acid in pregnancy on DNA methylation in the offspring, we carried out an epigenome-wide association study (EWAS) nested within the Aberdeen Folic Acid Supplementation Trial (AFAST). AFAST was a randomized–controlled trial of two different doses of folic acid (0.2 or 5 mg per day vs placebo) starting at booking for antenatal care at <30 weeks’ gestation that was performed in the late 1960s.^12–14^ Offspring of women who participated in AFAST, born during the trial, were identified and invited to participate in the present study at a mean age of 47 years. Their saliva samples were obtained for DNA-methylation profiling.

## Methods

### Parent study

#### Study design

AFAST has been described in detail elsewhere.^12–14^ Briefly, from June 1966 to June 1967, 3187 potentially eligible women (women booking for antenatal care at <30 weeks’ gestation who were resident in Aberdeen, UK) were invited to participate in a trial to examine the effects of folic-acid supplement use on pregnancy outcomes. Any woman for whom folic acid had been prescribed previously was excluded from the study.^13^ In all, 2928 women were randomized by alternate allocation to receive either 0.2 mg folic acid/day (*n* = 466, 15.6%), 5 mg folic acid/day (*n* = 485, 16.6%) or a placebo (*n* = 1977, 67.5%). Trial compliance was assessed by self-report and by measurement of folate status. In the placebo group, 1.9% reported that they had not taken their tablets regularly, compared with 1.7% in the group taking 0.2 mg folic acid and 3.2% in the group taking 5 mg. Prior to allocation, serum folate concentrations were similar in the three groups and a dose–response relationship was seen after allocation until the post-partum period, indicating that the tablets were regularly taken from the time of recruitment (mean gestational age at booking = 17 weeks) until the end of pregnancy (mean gestational age at delivery = 40 weeks).^14^ Among 2093 parous women, the incidence of a positive history of congenital malformation in a previous pregnancy was 2%.^13^

#### Baseline data collection

At the booking visit, the age of the mother, her gestation, parity, weight and blood pressure were recorded. The occupations of husbands/partners recorded on the study form at the time of delivery were used to determine the social class of the women based on the Classification of Occupations 1966.^15^ The trial database was linked to the Aberdeen Maternity and Neonatal Databank^16^ to add further demographic information on maternal smoking and height of mother. Additional information on mothers’ weight and blood pressure at booking were obtained from the original obstetric records. Serum folate was measured as previously described^14^ for 99.7% of women at the antenatal booking visit, 82.8% at approximately 30 weeks’ gestation, 37.2% at 36 weeks’ gestation and 63.4% in the postpartum period.

### Offspring study

#### Identification and recruitment of participants

Data from AFAST are archived within the Aberdeen Maternity and Neonatal Databank records held by the Institute of Applied Health Sciences, University of Aberdeen (http://www.abdn.ac.uk/iahs/research/obsgynae/amnd/index.php). For this study, the ‘affected’ offspring of trial participants (i.e. the children born during the mothers’ participation in the trial) were traced using the Community Health Index (CHI) and those living in the Grampian area were approached for participation by mail. Multiple births were excluded. A total of 692 offspring were invited to participate (Supplementary Figure 1, available as Supplementary data at *IJE* online) and sent an information leaflet and consent form.

#### Follow-up data collection

Participants who consented to participate (*N* = 265, Supplementary Figure 1, available as Supplementary data at *IJE* online) were mailed a short questionnaire to collect information on sex, age, self-reported height and weight, education, ethnicity, their health (e.g. current medications and health conditions) and current and past smoking status and alcohol intake. A saliva sample collection kit (Oragene, DNA Genetek, Kanata, Ontario, Canada) was provided and participants were asked to collect a saliva sample and return it through the post; 197 participants returned a saliva sample and 196 completed a questionnaire, representing a response rate of 28% (197/692).

The original trial treatment status of the study participant’s mother and other relevant trial data were provided by the Aberdeen Maternity and Neonatal Databank and linked to the offspring data. The linked anonymized data were given to researchers for analysis.

#### DNA methylation

Of the 196 individuals who returned a saliva sample and questionnaire, 180 individuals had DNA extracted from saliva that passed quality control (QC). Of these, 170 were female whereas only 10 were male. To minimize sex effects, we restricted profiling to females only and oversampled based on intervention status (111 individuals: 66 placebo, 21 low-dose folic acid, 24 high-dose folic acid). Genome-wide DNA-methylation profiling was performed on samples from 111 individuals using the Illumina Infinium HumanMethylation 450 array,^17^ run as described previously.^18^

**Figure 1:**
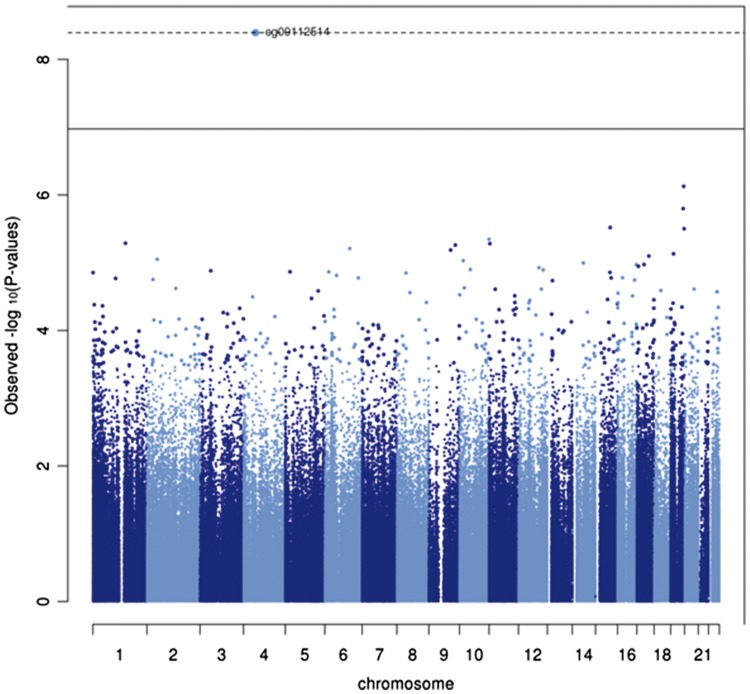
Manhattan plot for the EWAS of *in-utero* folic-acid supplement use (low and high dose combined vs placebo) (*N* = 86). Solid line = FDR threshold for association to account for multiple testing; Dotted line = Bonferroni corrected threshold for association to account for multiple testing.

Details of sampling handling and DNA-methylation profiling are outlined in the Supplementary Material, available as Supplementary data at *IJE* online. For this analysis, investigating the effect of intervention on methylation, we included 43 placebo and 43 intervention (20 low-dose and 23 high-dose) individuals to obtain a 1:1 placebo:intervention selection and reduce the effects of batch (Supplementary Material and Figure 1, available as Supplementary data at *IJE* online).

### Ethics approval

Ethics approval was given by the NRES Committee South West–Central Bristol REC. Approval to obtain addresses of the offspring through the CHI was obtained from the Caldicott Guardian, the Medical Director of NHS Grampian. This study was conducted in accordance with the Research Governance Framework for Health and Social Care and Good Clinical Practice and under the sponsorship of the University of Bristol. All samples were used and stored in accordance with the UK Human Tissue Act 2004.

### Statistical analysis

We first aimed to assess whether the baseline characteristics of the subset of individuals included in our analysis appeared to be equally distributed with regard to a number of maternal and offspring variables outlined earlier. Continuous baseline demographic characteristics across the three treatment groups were summarized as means and standard deviations, and tested for overall trend using one-way analysis of variance (ANOVA). Categorical baseline variables were summarized as percentages and numbers in each of the three treatment groups and an overall trend was tested by using the chi-squared test for trend.

#### EWAS

We next conducted an EWAS to investigate the long-term impact of the randomized folic-acid supplement-use intervention on offspring methylation in adulthood by evaluating the association between DNA methylation (normalized β value at 470 617 CpG sites on the array) and folic-acid supplement use.

We first combined both the 0.2- and 5-mg treatment groups to form a ‘folic acid supplement use’ group and carried out linear regression models to test the associations between the normalized β values at each CpG site as the dependent variable and folic-acid supplement use as the independent variable. Secondary analysis was then performed to determine associations between low-dose supplement use vs placebo, high-dose supplement use vs placebo and an ordinal model of high dose, low dose and placebo.

We adjusted for multiple testing using false discovery rate correction (FDR) and also investigated CpGs with a *p*-value <1×10^–5^. These analyses were adjusted for methylation array batch and also adjusted for gestational age at booking and age of the offspring at the time of data and sample collection in the main analysis (folic-acid supplement use vs placebo), given findings of a difference in these covariates between the treatment groups. Ten surrogate variables were generated using the ‘SVA’ package in R and included in models to adjust for technical batch and cell-type mixture^19^ given the absence of measured cell types in these samples. EWAS were performed using the ‘CpGassoc’ package^20^ implemented in R, which is designed to perform flexible analyses of methylation array data and to test for an association between methylation at CpG sites across the genome and phenotypes of interest, adjusting for relevant covariates. Sites were annotated using the information provided by Illumina.^9^

#### Regional approach

Adjacent probes on the HM450 array are often highly correlated and differentially methylated regions (DMRs) may be more biologically important than individual CpGs. Therefore, as well as our single-site (CpG) analysis, we also assessed differential methylation across larger regions of the genome in response to the intervention. For this, we used ‘Comb-P’ to identify regions enriched for low *p*-values, corrected for auto-correlation with neighbouring CpGs within 500 base pairs using the Stouffer-Liptak method and adjusted for multiple testing using the Sidak correction.^21^

#### Functional analysis

To explore the function of any identified DMRs, we used the missMethyl R package^22^ to test for enrichment for any gene ontology (GO) classification terms or the Kyoto Encyclopaedia of Genes and Genomes (KEGG) pathways. The method applies Fisher tests, while correcting for biases in the genomic coverage of the Illumina Infinium HumanMethylation450 BeadChip array. All CpGs on the array were used as background. We also used Fisher tests to test whether CpGs within our DMRs were enriched for CpGs within epialleles.^36^ Again, the background was all CpGs on the array. *P*-values for all enrichment analyses were adjusted for multiple testing using the FDR method.

#### Replication

For replication, we performed a look-up of epigenome-wide significant CpG sites from the single-site analysis in an EWAS meta-analysis of maternal plasma folate and DNA methylation (*N* = 1996) using summary-level data from this study obtained through dbGAP (dbGAP phs001059.v1.p1).^9^ Using these summary data from the EWAS meta-analysis, where effect estimates, standard errors and *p*-values were available for each CpG site, we also used Comb-P to identify overlap with the DMRs obtained from the EWAS in AFAST.

## Results

### Baseline characteristics

The baseline characteristics of the pregnant women in the three treatment groups were broadly comparable (Table 1), with the exception of gestational age at booking, where women in the high-dose (5 mg) folic-acid supplement group were enrolled at a later gestation than in the other two groups. However, this trend was not apparent in the larger sample of pregnant women in the trial (Supplementary Table 1, available as Supplementary data at *IJE* online),^14^^,^^23^ indicating that this difference is likely attributable to chance. An evaluation of baseline characteristics showed no clear differences between the mothers of offspring enrolled in this study compared with the original sample (Supplementary Table 1, available as Supplementary data at *IJE* online).

**Table 1:** Baseline characteristics of the mothers of participants in this study, collected as part of the original AFAST (1966–67) (*n* = 86)

Variable	Category	Placebo (*n* = 43)	Folic-acid supplement		*P*
			0.2 mg/day (*n* = 20)	5 mg/day (*n* = 23)	
**Categorical**		*n* (%)	*n* (%)	*n* (%)	Chi^2^
Age at delivery (years) (*N* = 86)	<20	5 (11.6)	3 (15.0)	4 (17.4)	
	20–24	10 (23.3)	8 (40.0)	10 (43.5)	
	25–29	16 (37.2)	4 (20.0)	5 (21.7)	
	≥30	12 (27.9)	5 (25.0)	4 (17.4)	0.50
Parity (*N* = 86)	0	16 (37.2)	5 (25.0)	11 (47.8)	
	1 or 2	19 (44.2)	12 (60.0)	9 (39.1)	
	≥3	8 (18.6)	3 (15.0)	3 (13.0)	0.53
Smoking in pregnancy (*N* = 83)	No	21 (51.2)	13 (68.4)	11 (47.8)	
	Yes	20 (48.8)	6 (31.6)	12 (52.2)	0.36
Social class (*N* = 85)	Non-manual	10 (23.3)	4 (20.0)	6 (27.3)	
	Manual	33 (76.7)	16 (80.0)	16 (72.7)	0.86
Pre-eclampsia (*N* = 86)	No	31 (72.1)	16 (80.0)	17 (73.9)	
	Mild	12 (27.9)	4 (20.0)	6 (26.1)	0.80
**Continuous**		Mean (SD)	Mean (SD)	Mean (SD)	ANOVA
BMI in pregnancy (kg/m^2^) (*N* = 84)		23.6 (3.2)	23.3 (3.7)	23.7 (3.2)	0.92
Gestational age at booking (weeks) (*N* = 86)		16.4 (4.3)	16.3 (4.5)	20.2 (5.9)	0.006
Serum folate at booking (ng/ml) (*N* = 86)		6.5 (3.3)	6.5 (3.0)	5.9 (3.3)	0.79

The characteristics of the female offspring from mothers in the three treatment groups were also similar (Table 2), with the exception of age at sample and data collection, whereby offspring in the placebo group were slightly older on average, and body mass index (BMI), which was higher in the intervention groups. Given these differences, we included gestational age and age at sample and data collection as covariates in subsequent models. As BMI may be a possible outcome or mediator of the intervention and methylation change, it was not considered as a covariate. An evaluation of baseline characteristics showed no clear differences between the offspring enrolled in this study compared with the original sample in terms of their birthweight or gestational age at delivery (Supplementary Table 1, available as Supplementary data at *IJE* online), although we were unable to assess differences between characteristics in adulthood, which were absent for those individuals who were not followed up.

**Table 2:** Characteristics of participants included in this study (AFAST offspring, *N* = 86)

Variable	Category	Placebo (*n* = 43)	Folic-acid supplement		
			0.2 mg/day (*n* = 20)	5 mg/day (*n* = 23)	
**Categorical**		*n* (%)	*n* (%)	*n* (%)	Chi^2^
Age at follow-up (years) (*N* = 86)	46	6 (14.0)	14 (70.0)	6 (26.1)	
	47	33 (76.7)	6 (30.0)	17 (73.9)	
	48	4 (9.3)	–	–	<0.001
Current smoking (*N* = 86)	No	26 (60.5)	9 (45)	14 (60.9)	
	Yes	17 (39.5)	11 (55)	9 (39.1)	0.47
Education (*N* = 84)	≤O-level	19 (46.3)	10 (50)	12 (52.2)	
	A-level/university	22 (53.7)	10 (50)	11 (47.8)	0.90
Alcohol intake	Daily/weekly	19 (44.2)	12 (60.0)	12 (52.2)	0.56
	Monthly	20 (46.5)	8 (40.0)	10 (43.5)	
	Not at all	4 (9.30)	0 (0)	1 (4.4)	
Folic-acid supplements	Yes	1 (2.3)	1 (5.0)	0 (0)	
	No	42 (97.7)	19 (95.0)	23 (100)	0.56
Current medication	Yes	31 (72.1)	15 (75.0)	17 (73.9)	0.97
	No	12 (27.9)	5 (25.0)	6 (26.1)	
Health problems	Yes	25 (58.1)	12 (60.0)	14 (60.9)	0.98
	No	18 (41.9)	8 (40.0)	9 (39.1)	
**Continuous**		Mean (SD)	Mean (SD)	Mean (SD)	ANOVA
BMI (kg/m^2^) (*N* = 85)		24.2 (3.8)	26.5 (6.4)	27.8 (7.2)	0.04
Length of gestation (weeks) (*N* = 86)		40.9 (1.1)	39.9 (2.6)	40.3 (1.5)	0.07
Birthweight (g) (*N* = 86)		3333 (506)	3093 (620)	3269 (493)	0.25

### EWAS

**Figure 2:**
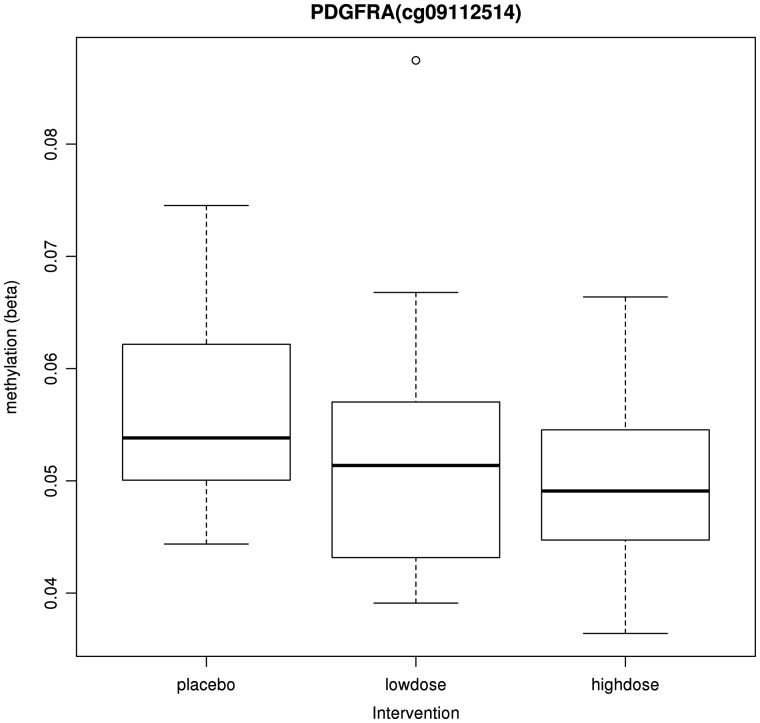
Box plot for methylation at *PDGFRA* (cg09112514) in the different intervention groups (*N* = 86).

We next conducted an EWAS to investigate the long-term impact of the randomized folic-acid supplement-use intervention on offspring methylation in adulthood by evaluating the association between DNA methylation (normalized β value at each of the 460 617 CpG sites on the array) and folic-acid supplement use. We found an association at just one CpG site, cg09112514, which withstood the FDR correction where the intervention was associated with a 0.8% [95% confidence interval (CI) = 0.4, 1.2] reduction in methylation at this site (Figure 1). A further 14 CpG sites were found to surpass a less conservative *p*-value threshold of 1×10^–5^ and effects were generally not attenuated with additional adjustment for gestational age and age at follow-up as covariates (Supplementary Table 2, available as Supplementary data at *IJE* online). We observed a reduction in methylation at 9 of these 14 CpG sites in the folic-acid supplement group vs placebo. Furthermore, the same CpG site, cg09112514, was found to be most strongly associated with both the low and high doses when considered in separate models and was also strongly associated in the ordinal model of high dose, low dose and placebo (*p* = 4.47×10^–7^) (Supplementary Tables 3–5, available as Supplementary data at *IJE* online) and illustrated a dose–response with regard to the intervention arm (Figure 2).

We next investigated whether any of the CpG sites that surpassed the *p*-value threshold of 1×10^–5^ were identified as being either single-nucleotide polymorphism (SNP)-confounded or cross-hybridizing based on a comprehensive assessment reported by Naeem *et al.*^24^ Two CpG sites, cg25455598 and cg13682325, identified in the main analysis were flagged by this study as lower-quality probes (Supplementary Table 2, available as Supplementary data at *IJE* online).

### DMRs

Given the low power available in this study of just 86 individuals to identify strong site-specific signals, we considered taking a regional approach to assess DMRs of the genome in response to the intervention. This was further supported by the Q-Q and Volcano plots of the site-specific EWAS analysis that showed an inflation of *p*-values above that expected by chance in the main analysis of intervention vs placebo and particularly for the high dose vs placebo model (Supplementary Figure 2, available as Supplementary data at *IJE* online). In the DMR analysis, we identified 46 DMRs with a Sidak *p*-value (multiple testing corrected) < 0.05 in the main model (folic-acid supplement use vs placebo) (Supplementary Table 6 and Figure 3a, available as Supplementary data at *IJE* online). Furthermore, for the high dose vs placebo model and low dose vs placebo model, 28 DMRs (Supplementary Table 7 and Figure 3b, available as Supplementary data at *IJE* online) and 2 DMRs (Supplementary Figure 3c and Table 8, available as Supplementary data at *IJE* online) were identified, respectively. Notable regions included *HLA-DPB2* and *HLA-DPB1*, which had low regional *p*-values in all models; *PAX8*, which was found to have low regional *p*-values in both the main and high-dose models; and *VTRNA2–1*, which was found to have the lowest regional *p*-value in the high-dose model.

### Functional analysis

CpGs within DMRs identified using the main model (folic-acid supplement use vs placebo; 303 CpGs; Supplementary Table 6, available as Supplementary data at *IJE* online) were most enriched for KEGG pathways relating to cancer and regulation of the actin cytoskeleton (FDR-adjusted *p*-value for enrichment = 0.002) and GO terms related to kidney development, although it should be noted that no GO terms were enriched after correction for multiple testing. Similarly, CpGs within DMRs identified using the high- and low-dose models were not enriched for any KEGG pathways or GO terms after FDR correction (Supplementary Tables 9 and 10, available as Supplementary data at *IJE* online).

CpGs within DMRs identified using the main model (303 CpGs; Supplementary Table 6, available as Supplementary data at *IJE* online) were highly enriched for CpGs within epiallelic regions (133 CpGs; Supplementary Table 11, available as Supplementary data at *IJE* online): six CpGs within DMRs were also within epiallelic regions (Chi-Square 353.89; *p* = 3.6×10^–10^). All six CpGs were within a DMR mapping to *PAX8* (Chr2: 113992762–113993314). CpGs within DMRs identified using the high-dose model (high dose vs placebo; 170 CpGs; Supplementary Table 7, available as Supplementary data at *IJE* online) were also enriched for epialleles: 16 CpGs within DMRs were within epiallelic regions (Chi-square 5131.91; *p* = 4.3×10^–36^). Six out of 16 CpGs were in a DMR mapping to *PAX8* and the remaining 10 CpGs were in a DMR mapping to VTRNA2–1 (Chr5: 135414858–135416614).

### Replication

Using summary findings from a neonatal EWAS of maternal plasma folate, we performed a look-up of cg09112514 (*PDGFRA*) and found that this did not replicate in that study (*p* = 0.96). We also performed a DMR analysis of EWAS summary findings and then identified overlap between the DMRs identified in AFAST. A DMR at *PAX8* with a regional *p*-value of 2.08×10^–6^ in AFAST had a regional *p*-value of 2.46×10^–10^ in this independent replication sample (Supplementary Figure 4 and Table 12, available as Supplementary data at *IJE* online).

## Discussion

The results of this study, conducted within the context of a randomized–controlled trial, suggest that maternal folic-acid supplement use is associated with changes in DNA methylation that persist for many years after *in-utero* exposure. In saliva samples obtained from the offspring 47 years after the trial was conducted, an effect of folic-acid supplement use on DNA methylation was identified at cg09112514 (*p* = 4.03×10^–9^), a CpG site in the 5’ UTR of *PDGFRA*, in the main single-site EWAS analysis comparing the intervention arms [low (0.2 mg) and high (5 mg) dose folic acid] (*N* = 43) vs placebo (*N* = 43). Furthermore, a dose–response reduction in methylation at this site was identified with regard to the intervention. We also identified 46 regions of the genome that were differentially methylated in response to folic-acid supplement use, including *HLA-DPB2*, *HLA-DPB1*, *PAX8* and *VTRNA2–1*.

*PDGFRA* encodes a platelet-derived growth factor receptor that has been linked with congenital neural tube defects (NTDs) and isolated cleft palate.^25^ In particular, mouse models have indicated that deregulated expression of this gene leads to NTD formation^26^ and specific haplotypes of the *PDGFRA* P1 promoter strongly affect rates of NTD genesis.^27^^,^^28^ Furthermore, methylation in this gene region has recently been linked with subtypes of orofacial cleft.^29^ It is therefore interesting that we identified differential methylation at a site in *PDGFRA* in relation to folic-acid supplement use, given the well-established link between folate status in pregnancy and risk of such birth defects.^30–32^ Although the effect size was small (0.8% reduction in methylation at this site in the folic-acid supplement-use group), this does not preclude biological plausibility of this methylation difference, which may have subtle effects on health outcomes.

Differences in genome-wide DNA methylation have been evaluated in relation to maternal folate and other micronutrient exposures in candidate gene studies^33^ and EWAS.^34^ However, unlike the study conducted here, most previous studies investigating maternal folate have measured methylation only in newborn infants,^34^ with just one study^35^ evaluating methylation at a later time point in infancy. Therefore, our study is novel in investigating methylation change into adulthood in relation to this prenatal exposure.

Nevertheless, we attempted to replicate our findings in the largest EWAS of maternal folate conducted to date (*N* = 1996).^9^ We found no clear association between maternal cg09112514 and maternal plasma folate levels in this study. This lack of replication between studies may reflect methylation profiling in different tissues (saliva vs cord blood), differences in the timing of methylation assessment (adults vs newborn infants), differences in the exposure measure (folic-acid supplement use vs maternal plasma folate) or other differences in the study design and populations investigated.^34^ Alternatively, the lack of replication might indicate that this signal represents a false-positive finding, given the small sample size of our study.

To combat the low power in our EWAS, we also took a regional approach to assess DMRs of the genome in response to the intervention. We also assessed replication of the DMRs in the results from the previous EWAS^9^ and this time found some suggested replication of differential methylation at *PAX8* in relation to maternal folate. In further support for the robustness of the DMR findings, both *PAX8* and *VTRNA2–1* are notable, as they are gene regions in which deemed ‘metastable epialleles’ have previously been identified in relation to peri-conceptional nutrition.^11^^,^^36^ Metastable epialleles are defined as those that are influenced by the *in-utero* environment, occur systemically and are highly stable over many years,^11^ which are reflective of the methylation changes observed in this study. However, whereas these previous studies highlight the importance of the periconceptional environment for establishing methylation marks at these metastable epialleles, in this study, the intervention was initiated at an average gestational age of 16 weeks. Similarly, our top site in *PDGFRA* is implicated in NTDs but the critical period for folate status on risk of NTDs is thought to be periconceptional. Nonetheless, our results are consistent with previous findings suggesting that environmentally induced DNA-methylation change may not be limited to the periconceptional period.^37^

We observed a reduction in methylation at 10 of the 15 top CpG sites in the folic-acid supplement use vs placebo groups (Supplementary Table 2, available as Supplementary data at *IJE* online) as well as a reduction in methylation at 287 of the 303 CpG sites contributing to the top DMRs in the folic-acid supplement use vs placebo groups (Supplementary Table 6, available as Supplementary data at *IJE* online). Furthermore, there was widespread (although low-magnitude) hypomethylation among those CpGs not surpassing multiple testing correction in response to the intervention (Supplementary Figure 2, available as Supplementary data at *IJE* online). These findings of hypomethylation in relation to folic-acid exposure are consistent with previous findings,^8^^,^^9^^,^^38^ despite the fact that folate is a methyl donor (and therefore might be anticipated to increase methylation levels at these CpG sites). Nonetheless, as was discussed previously,^9^ folic acid has been shown to disturb the intracellular one-carbon metabolism by inhibiting methylenetetrahydrofolate reductase (MTHFR) activity that may decrease DNA methylation^39^ and so our findings are not inconsistent with respect to known biological pathways.

Key strengths of this study include the experimental design in which this study was nested, with random allocation, adequate concealment and evidence of good compliance.^14^ In addition, given the identified role of folic-acid supplement use in the prevention of NTDs, an RCT to determine the long-term effects of *in-utero* exposure to folic acid vs placebo would no longer be ethical. This historical study therefore provides a unique opportunity to investigate the impact of folic-acid supplements in pregnancy on long-term DNA-methylation changes in a trial setting. It also illustrates a successful attempt of enrolling individuals into a study through data-record linkage approximately 47 years after the initial trial, which allowed us to look at long-term effects of *in-utero* exposure to folic acid. Furthermore, participants included in this study were similar to the original study sample with respect to the baseline characteristics, indicating that the randomized nature of the intervention was preserved. This study also highlights the value of saliva as a non-invasive sample on which to perform DNA-methylation profiling^40^ and the value of methylation profiles as a biosocial archive for historical exposure.^41^

Limitations include the small sample size of this study, which might have generated spurious associations at the single-CpG level in the EWAS as a result of low power, although the replication of DMRs in independent studies supports the robustness of these findings. A further limitation relates to the fact that this study was conducted in female offspring only and individuals residing within the Grampian area, meaning results may not be generalizable. As the offspring response rate was 28%, selection bias could have played a role in our results, although an evaluation of baseline characteristics revealed no clear differences between the mothers or offspring of those enrolled in this study compared with the original sample.

Overall, the results of this study suggest that maternal folic-acid supplement use, even after the periconceptional period, is associated with changes in DNA methylation in the offspring that persist for many years after *in-utero* exposure. These methylation changes are located in genes implicated in pathways related to cancer, metabolism and infection, and therefore may mediate long-term effects of folic-acid exposure in pregnancy.^14^^,^^42^^,^^43^ However, the causal relevance of these methylation changes with regard to these developmental and health outcomes remains to be determined. Further work to investigate whether these epigenetic changes translate into detectable phenotypic differences is required. 

## Supplementary data

Supplementary data are available at *IJE* online

## Funding

This work was supported by the NIHR Bristol Biomedical Research Centre at the University Hospitals Bristol NHS Foundation Trust and the University of Bristol. The views expressed in this publication are those of the authors and not necessarily those of the NHS, the National Institute for Health Research or the Department of Health. R.C.R., G.C.S., N.K., T.G., G.D.S. and C.L.R. work in a unit that receives funds from the University of Bristol and the UK Medical Research Council (MC_UU_12013/1, MC_UU_12013/2 and MC_UU_12013/8). This work was also supported by CRUK (grant number C18281/A19169) and the ESRC (grant number ES/N000498/1). C.M.T. is supported by a Wellcome Trust Career Re-entry Fellowship (grant number 104077/Z/14/Z).

**Conflict of interest:** None declared.

## Supplementary Material

Supplementary Data
