# Supplementary material for: The long-term impact of folic acid in pregnancy on offspring DNA methylation: follow-up of the Aberdeen Folic Acid Supplementation Trial (AFAST)
Source: Int J Epidemiol. 2018 Mar 12;47(3):928–37. doi: 10.1093/ije/dyy032 (PMC6005053; doi:10.1093/ije/dyy032)
Supplement: Supplementary Data [file dyy032_supp.zip › dyy032-suppl_data/ije-2017-05-0586-File005.docx]

## Methods

### Sample handling and DNA methylation profiling

DNA was extracted from saliva samples and bisulphite-converted using the Zymo EZ DNA MethylationTM kit (Zymo, Irvine, CA) according to standard procedures. Samples were loaded onto the Illumina Infinium HumanMethylation 450 array in three batches and processed blind to sample identification in the University of Bristol Bioresource Laboratories.

Raw data were converted into beta-values (β) that ranged from 0 (unmethylated) to 1 (fully methylated) and Functional Normalization was employed using the *minfi* package in R to normalize data (1). In order to exclude low quality samples, raw methylation data QC was performed. First, CpG detection p-values were calculated for all samples: those with an average p-value of >0.01 were termed as failed (undetectable) samples and were excluded prior to any further QC checks. We verified the intensities of several control probes provided and outliers were excluded. We also filtered out methylation probes with a detection p value >0.01 in at least 1% of samples and omitted probes on the X chromosome. In total, 460,617 CpGs remained after these exclusions.

To assess technical replication, we also investigated correlation between four duplicate samples run in different batches on the methylation array. To do this, we first scaled the methylation matrix (to identify variability among samples rather than among CpG sites), winsorized to exclude outliers, and compared median correlation between technical replicate pairs with the median correlation between all pairwise comparisons in the dataset, using the Wilcoxon rank sum test as a test for difference. Some evidence for a difference between correlations of the technical replicate pairs with the correlations between all pairwise comparisons in the dataset indicated adequate concordance of four duplicate samples (median *r* (after scaling) = 0.15 for technical replicates, median *r* (after scaling) = -0.01 for all pairwise comparisons; p-value for difference = 0.04).

Samples from 108 females passed QC. Because we oversampled individuals who received intervention in early batches there was an uneven distribution of intervention groups which could induce large batch effects. For this analysis investigating the effect of intervention on methylation, we excluded (at random) 22 placebo samples from the third batches, leaving 43 placebo and 43 intervention (20 low dose and 23 high dose) individuals to obtain a 1:1 placebo : intervention selection and reduce the effects of batch (chi^2^ P = 0.255) (**S1 Figure**).

References

1. Aryee MJ, Jaffe AE, Corrada-Bravo H, Ladd-Acosta C, Feinberg AP, Hansen KD, et al. Minfi: a flexible and comprehensive Bioconductor package for the analysis of Infinium DNA methylation microarrays. Bioinformatics. 2014;30(10):1363-9.
