# Supplementary material for: The long-term impact of folic acid in pregnancy on offspring DNA methylation: follow-up of the Aberdeen Folic Acid Supplementation Trial (AFAST)
Source: Int J Epidemiol. 2018 Mar 12;47(3):928–37. doi: 10.1093/ije/dyy032 (PMC6005053; doi:10.1093/ije/dyy032)
Supplement: Supplementary Data [file dyy032_supp.zip › dyy032-suppl_data/ije-2017-05-0586-File006.docx]

|  | Selected individuals (n=86) | | | Original sample (n=2,928) | | |
| --- | --- | --- | --- | --- | --- | --- |
|  | Placebo  (n=43) | Folic acid 200µg/day  (n=20) | Folic acid 5 mg/day (n=23) | Placebo (n=1,977) | Folic acid 200µg/day  (n=466) | Folic acid 5 mg/day  (n=485) |
| Variable | Mean (SD) | | | | | |
| Age (years) | 27.0 (5.5) | 26.8 (7.1) | 23.8 (5.3) | 26.0 (5.6) | 25.8 (5.5) | 25.5 (5.2) |
| GA at booking (weeks) | 16.4 (4.3) | 16.3 (4.5) | 20.2 (5.9) | 17.5 (5.3) | 16.9 (4.8) | 17.7 (5.6) |
| Weight at booking (kg) | 59.9 (8.1) | 58.3 (10.4) | 59.9 (8.1) | 59.6 (9.5) | 59.0 (8.9) | 59.6 (9.1) |
| Height (cm) | 158.7 (5.1) | 158.0 (5.8) | 159.2 (7.1) | 159.5 (5.9) | 159.4 (6.4) | 159.5 (6.1) |
| Birthweight crude (g) | 3333 (506) | 3093 (620) | 3269 (483) | 3299 (512) | 3286 (528) | 3319 (473) |
| GA at delivery (weeks) | 40.9 (1.1) | 39.9 (2.6) | 40.3 (1.5) | 40.2 (1.9) | 40.1 (2.0) | 40.3 (1.7) |
|  | N (%) | | | | | |
| Smoked at booking | 20 (46.5) | 6 (30.0) | 12 (52.2) | 840 (45.8) | 188 (42.2) | 203 (44.7) |
| Social class code |  |  |  |  |  |  |
| I | 1 (2.3) | 0 (0) | 0 (0) | 122 (6.2) | 31 (6.7) | 34 (7.0) |
| II | 3 (7.0) | 2 (10.0) | 2 (9.1) | 219 (11.1) | 44 (9.4) | 49 (10.1) |
| IIIN | 6 (14.0) | 2 (10.0) | 4 (18.2) | 204 (10.3) | 61 (13.1) | 63 (13.0) |
| IIIM | 20 (46.5) | 11 (55.0) | 9 (40.9) | 768 (38.8) | 170 (36.5) | 146 (29.9) |
| IV | 6 (14.0) | 1 (5.0) | 5 (22.7) | 330 (16.7) | 70 (15.0) | 91 (18.8) |
| V | 7 (16.3) | 4 (20.0) | 1 (4.6) | 164 (8.3) | 43 (9.2) | 44 (9.1) |
| Undefined or armed forces | 0 (0) | 0 (0) | 1 (4.6) | 170 (8.6) | 47 (10.1) | 59 (12.2) |
| Parity |  |  |  |  |  |  |
| 0 | 16 (37.2) | 5 (25.0) | 11 (47.8) | 734 (37.1) | 198 (42.5) | 196 (40.4) |
| 1-3 | 23 (53.5) | 14 (70.0) | 10 (43.5) | 904 (45.7) | 216 (46.4) | 224 (46.2) |
| >=4 | 4 (9.3) | 1 (5.0) | 2 (8.7) | 339 (17.1) | 52 (11.2) | 65 (13.4) |
|  |  |  |  |  |  |  |

S1 Table – Characteristics of individuals and mothers of individuals included in this study (AFAST offspring, n=86), compared with those in the original sample from the Aberdeen Folic Acid Supplementation Trial (1966-1967) (n=2,928)
