# Supplementary material for: The long-term impact of folic acid in pregnancy on offspring DNA methylation: follow-up of the Aberdeen Folic Acid Supplementation Trial (AFAST)
Source: Int J Epidemiol. 2018 Mar 12;47(3):928–37. doi: 10.1093/ije/dyy032 (PMC6005053; doi:10.1093/ije/dyy032)
Supplement: Supplementary Data [file dyy032_supp.zip › dyy032-suppl_data/ije-2017-05-0586-File007.docx]

**S2 Table** EWAS results for the main model of in utero folic acid supplement use (low and high dose combined vs placebo) (P < 1 x 10-5)

|  |  |  |  | Basic model *(N=86) | | | Adjusted model ^±^(N= 86) | | |
| --- | --- | --- | --- | --- | --- | --- | --- | --- | --- |
| CpG site | Chromosome | Gene region | Position | Effect size (β value) | Standard error | P-value | Effect size (β value) | Standard error | P-value |
| cg09112514 | 4 | *PDGFRA* | 55096230 | -0.008 | 0.001 | 4.03X10-9 | -0.007 | 0.001 | 5.81X10-6 |
| cg15241920 | 19 | *TTYH1* | 54926514 | -0.004 | 0.001 | 7.46X10-7 | -0.004 | 0.001 | 2.34X10-5 |
| cg17506458 | 19 |  | 52602607 | -0.024 | 0.005 | 1.59X10-6 | -0.021 | 0.006 | 5.55X10-4 |
| cg07057074 | 15 | *MIR548H4;GLCE* | 69452784 | -0.002 | 0.0004 | 3.03X10-6 | -0.002 | 0.002 | 1.73X10-4 |
| cg18787401 | 19 | *ZNF542* | 56879559 | -0.004 | 0.001 | 3.16X10-6 | -0.005 | 0.001 | 6.68X10-5 |
| cg26182964 | 10 | *ECHS1* | 1.35E+08 | 0.040 | 0.008 | 4.50X10-6 | 0.044 | 0.010 | 2.58X10-5 |
| cg00785522 | 1 | *ADAMTSL4* | 1.51E+08 | -0.004 | 0.001 | 5.16X10-6 | -0.005 | 0.001 | 1.28X10-6 |
| cg25455598^‡^ | 11 | *PGAP2* | 3829824 | -0.001 | 0.0003 | 5.23X10-6 | -0.001 | 0.0004 | 2.85X10-4 |
| cg13682325^‡^ | 9 | *DBC1* | 1.22E+08 | 0.049 | 0.010 | 5.50X10-6 | 0.040 | 0.012 | 1.32X10-3 |
| cg11128944 | 6 |  | 1.14E+08 | 0.029 | 0.006 | 6.18X10-6 | 0.028 | 0.007 | 3.58X10-4 |
| cg14379939 | 9 |  | 1.01E+08 | -0.026 | 0.005 | 6.51X10-6 | -0.022 | 0.007 | 1.12X10-3 |
| cg18394178 | 19 | *CACNA1A* | 13397644 | 0.034 | 0.007 | 7.40X10-6 | 0.032 | 0.009 | 3.94X10-4 |
| cg19872368 | 17 | *YPEL2* | 57409544 | -0.002 | 0.0004 | 7.97X10-6 | -0.002 | 0.001 | 3.97X10-4 |
| cg11253886 | 2 |  | 47039669 | 0.044 | 0.009 | 8.91X10-6 | 0.046 | 0.011 | 7.69X10-5 |
| cg12874092 | 10 | *VIM* | 17271519 | -0.002 | 0.0003 | 9.33X10-6 | -0.002 | 0.0004 | 2.70X10-6 |

* Adjusted for 10 SVs only ^±^Adjusted for 10 SVs, offspring gestational age at birth and age at follow-up ^‡^Naeem flagged CpG site
