# Supplementary material for: The long-term impact of folic acid in pregnancy on offspring DNA methylation: follow-up of the Aberdeen Folic Acid Supplementation Trial (AFAST)
Source: Int J Epidemiol. 2018 Mar 12;47(3):928–37. doi: 10.1093/ije/dyy032 (PMC6005053; doi:10.1093/ije/dyy032)
Supplement: Supplementary Data [file dyy032_supp.zip › dyy032-suppl_data/ije-2017-05-0586-File008.docx]

**S3 Table** - EWAS results of in utero folic acid supplement use (high dose vs placebo) (P < 1 x 10-5)

|  |  |  |  | Basic model *(N= 66) | | |
| --- | --- | --- | --- | --- | --- | --- |
| CpG site | Chromosome | Gene region | Position | Effect size | Standard error | P-value |
| cg09112514 | 4 | *PDGFRA* | 55096230 | -0.008 | 0.001 | 1.42X10-6 |
| cg24598330 | 12 | *NR2C1* | 93991481 | -0.004 | 0.000793 | 2.32X10-6 |
| cg00785522 | 1 | *ADAMTSL4* | 1.49E+08 | -0.005 | 0.000888 | 2.85X10-6 |
| cg02539809 | 7 | *C7orf58* | 1.21E+08 | 0.029 | 0.005675 | 5.34X10-6 |
| cg17506458 | 19 | *ZNF841* | 57294419 | -0.029 | 0.005364 | 5.75X10-6 |
| cg19625524 | 4 | *FGFR3* | 1772946 | 0.003 | 0.000687 | 5.97X10-6 |
| cg15982419 | 18 | *RALBP1* | 9464707 | -0.080 | 0.01598 | 6.09X10-6 |
| cg08479635 | 11 | *PRDM11* | 45071146 | -0.028 | 0.00567 | 6.48X10-6 |
| cg14213844 | 20 | *BLCAP* | 35590059 | -0.001 | 0.00024 | 6.60X10-6 |
| cg25713684 | 10 | *TAF5* | 1.05E+08 | -0.008 | 0.001649 | 7.35X10-6 |
| cg00870662 | 18 | *PARD6G* | 76106656 | -0.003 | 0.000652 | 8.45X10-6 |
| \| cg11128944 \| \| --- \| | 6 | *FLJ34503* | 1.14E+08 | 0.033 | 0.006673 | 9.12X10-6 |
| cg06899192 | 17 | *TADA2A* | 32841177 | -0.004 | 0.000768 | 9.32X10-6 |

* Adjusted for 10 SVs only
