# Supplementary material for: The long-term impact of folic acid in pregnancy on offspring DNA methylation: follow-up of the Aberdeen Folic Acid Supplementation Trial (AFAST)
Source: Int J Epidemiol. 2018 Mar 12;47(3):928–37. doi: 10.1093/ije/dyy032 (PMC6005053; doi:10.1093/ije/dyy032)
Supplement: Supplementary Data [file dyy032_supp.zip › dyy032-suppl_data/ije-2017-05-0586-File009.docx]

**S4 Table** - EWAS results of in utero folic acid supplement use (low dose vs placebo) (P < 1 x 10-5)

|  |  |  |  | Basic model *(N= 66) | | |
| --- | --- | --- | --- | --- | --- | --- |
| CpG site | Chromosome | Gene region | Position | Effect size | Standard error | P-value |
| cg09112514 | 4 | *PDGFRA* | 55096230 | -0.008 | 0.002 | 1.42X10-6 |
| cg18787401 | 19 | *ZNF542* | 56879559 | -0.006 | 0.001 | 5.15X10-6 |
| cg11102098 | 10 | *KIAA1598* | 1.19E+08 | 0.041 | 0.008 | 6.46X10-6 |
| cg17828223 | 6 | *FBXO5* | 1.53E+08 | -0.001 | 0.0002 | 7.80X10-6 |
| cg00373101 | 16 | *ITPRIPL2* | 19125146 | -0.017 | 0.004 | 9.73X10-6 |

* Adjusted for 10 SVs only
