# Supplementary material for: The long-term impact of folic acid in pregnancy on offspring DNA methylation: follow-up of the Aberdeen Folic Acid Supplementation Trial (AFAST)
Source: Int J Epidemiol. 2018 Mar 12;47(3):928–37. doi: 10.1093/ije/dyy032 (PMC6005053; doi:10.1093/ije/dyy032)
Supplement: Supplementary Data [file dyy032_supp.zip › dyy032-suppl_data/ije-2017-05-0586-File010.docx]

**S5 Table** - EWAS results of in utero folic acid supplement use (ordinal model: high dose, low dose and placebo) (P < 1 x 10-5)

|  |  |  |  | Basic model *(N= 86) | | |
| --- | --- | --- | --- | --- | --- | --- |
| CpG site | Chromosome | Gene region | Position | Effect size | Standard error | P-value |
| cg00785522 | 1 | *ADAMTSL4* | 150521766 | -0.003 | 0.001 | 2.54E-07 |
| cg09112514 | 4 | *PDGFRA* | 55096230 | -0.004 | 0.001 | 4.47E-07 |
| cg24598330 | 12 | *NR2C1* | 95467350 | -0.002 | 0.0004 | 4.74E-06 |
| cg17506458 | 19 | *ZNF841* | 52602607 | -0.013 | 0.003 | 4.82E-06 |
| cg11128944 | 6 | *FLJ34503* | 114215021 | 0.018 | 0.004 | 6.44E-06 |
| cg06899192 | 17 | *ACACA* | 35767064 | -0.002 | 0.0003 | 7.43E-06 |
| cg07301433 | 2 | *CYP1B1* | 38303999 | -0.001 | 0.0002 | 7.53E-06 |

* Adjusted for 10 SVs only
