# Supplementary material for: The long-term impact of folic acid in pregnancy on offspring DNA methylation: follow-up of the Aberdeen Folic Acid Supplementation Trial (AFAST)
Source: Int J Epidemiol. 2018 Mar 12;47(3):928–37. doi: 10.1093/ije/dyy032 (PMC6005053; doi:10.1093/ije/dyy032)
Supplement: Supplementary Data [file dyy032_supp.zip › dyy032-suppl_data/ije-2017-05-0586-File017.docx]

**S1 Figure** - Flow diagram for Aberdeen Folic Acid Supplementation Trial offspring study

*Because we oversampled individuals who received intervention in early batches there was an uneven distribution of intervention groups which could induce large batch effects. For the purposes of the analysis investigating effect of intervention on methylation, we excluded (at random) 22 placebo samples from Batch 3, leaving 43 placebo and 43 intervention (20 low dose and 23 high dose) to reduce the effect of batch.
