# Supplementary material for: The long-term impact of folic acid in pregnancy on offspring DNA methylation: follow-up of the Aberdeen Folic Acid Supplementation Trial (AFAST)
Source: Int J Epidemiol. 2018 Mar 12;47(3):928–37. doi: 10.1093/ije/dyy032 (PMC6005053; doi:10.1093/ije/dyy032)

**S2 Figure** – QQ and Volcano plots for the EWAS results

a) Folic acid supplement use (low and high dose combined) vs. placebo


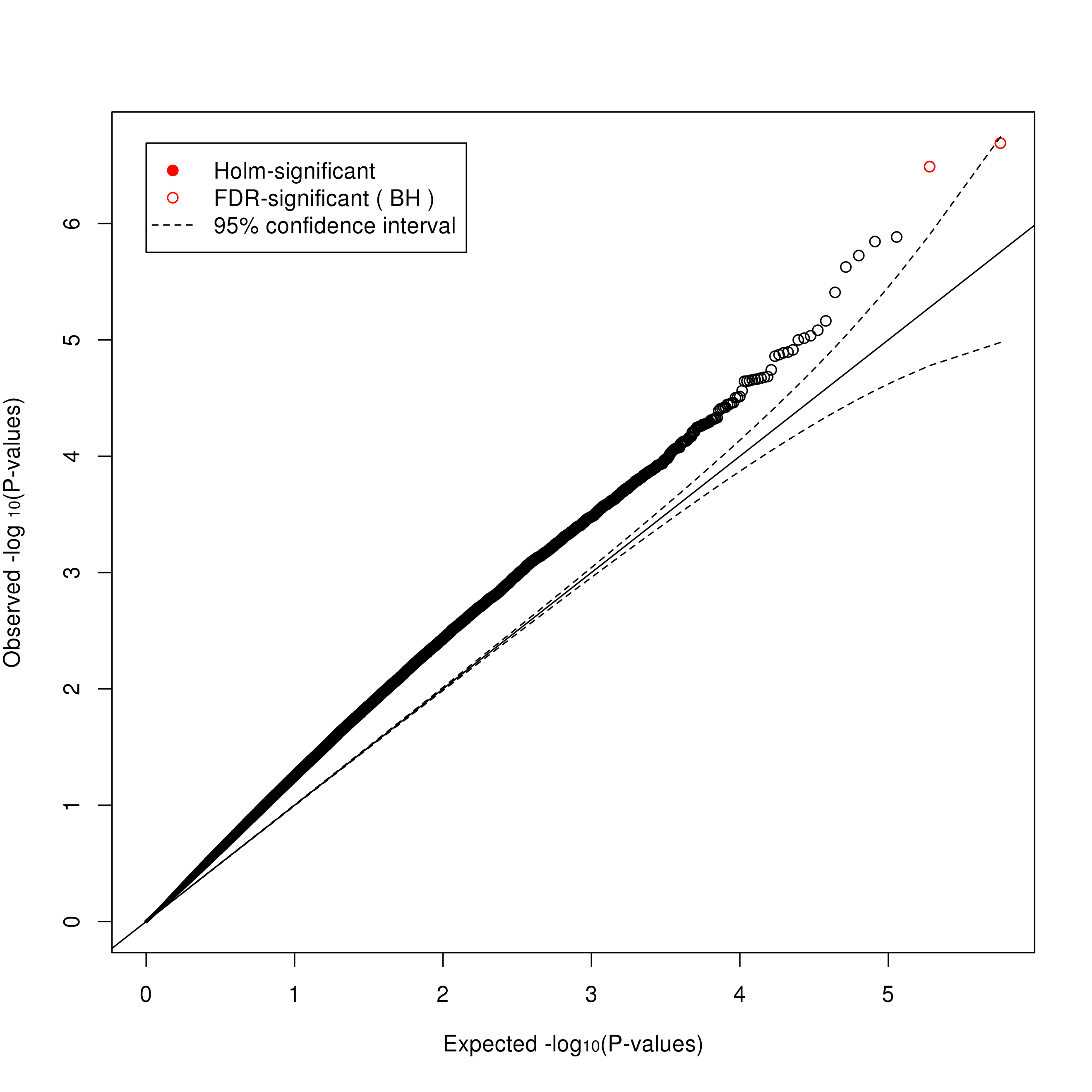

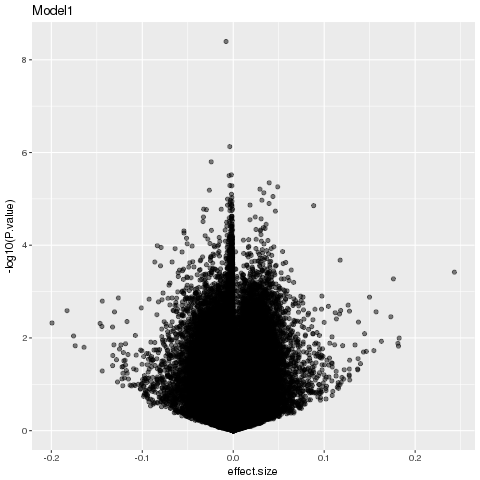


b) High dose vs. placebo


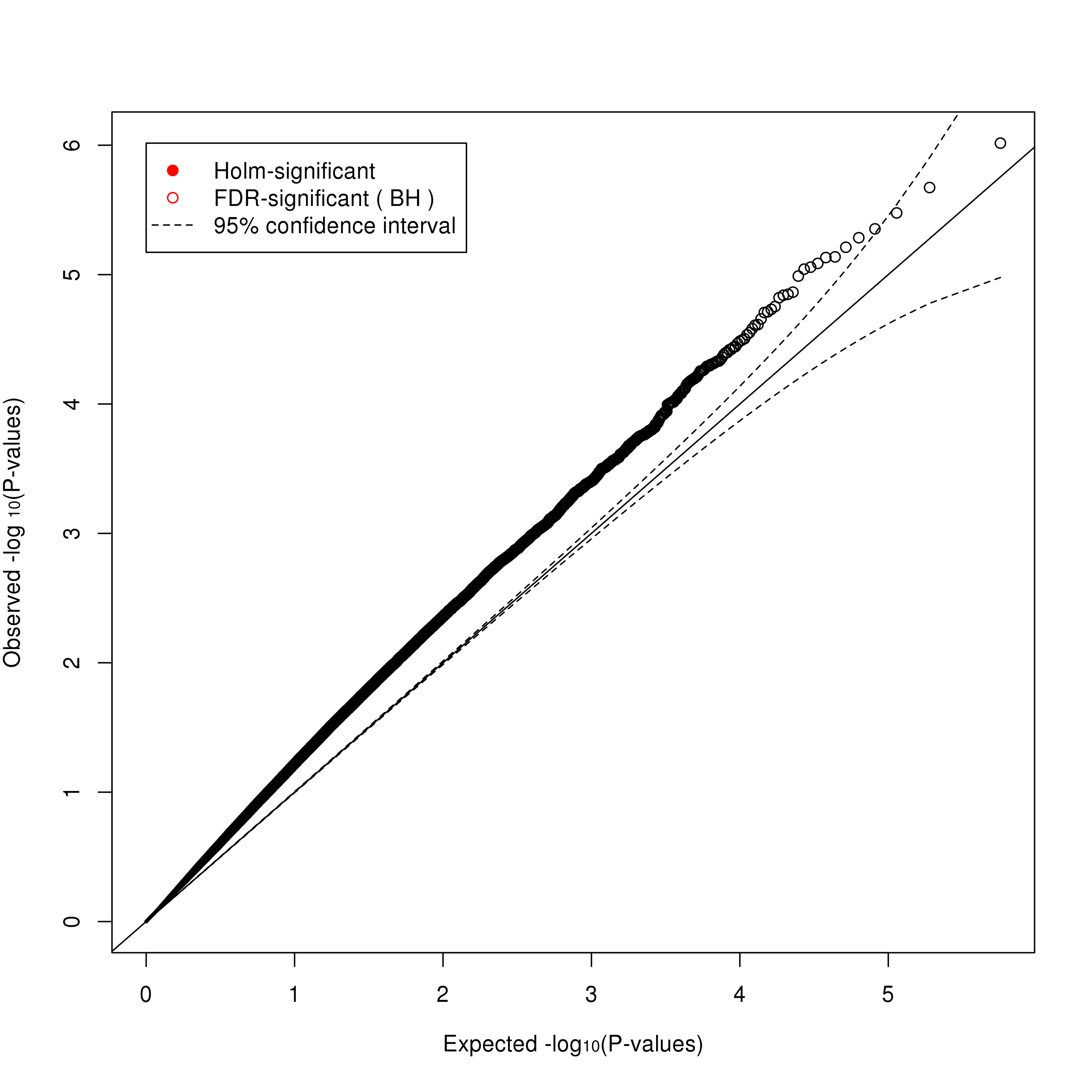

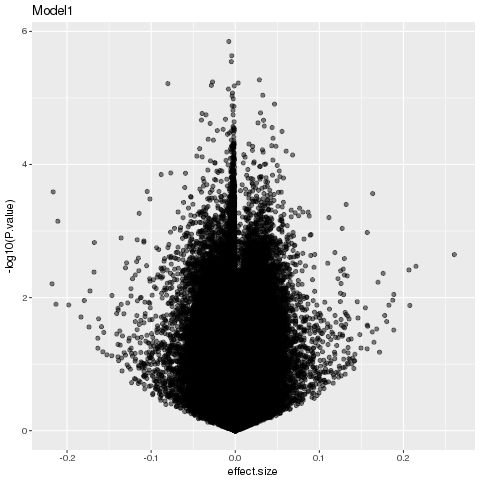


c) Low dose vs. placebo


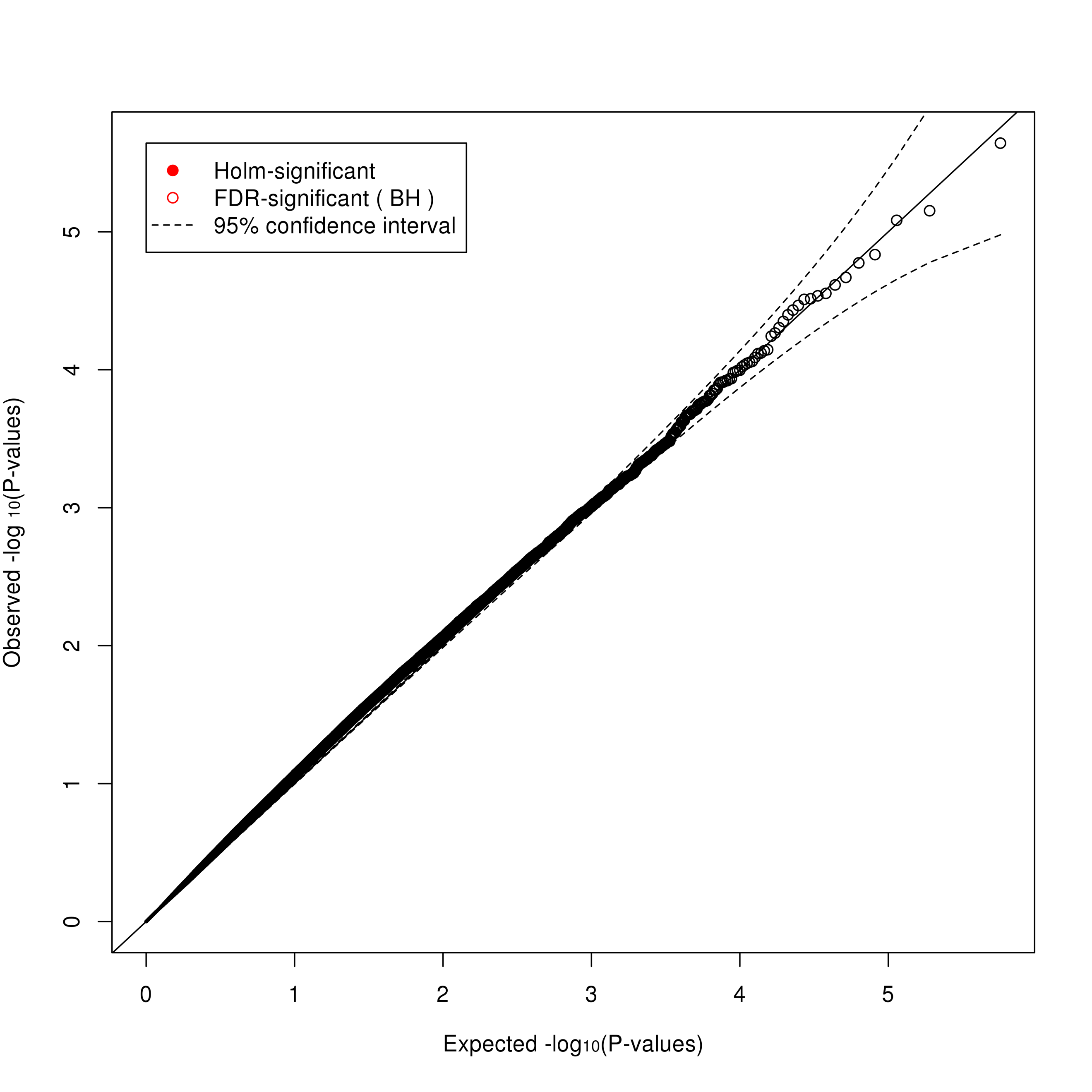

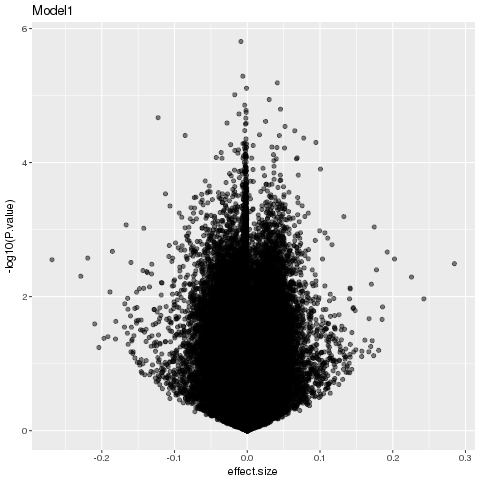

Supplement: Supplementary Data [file dyy032_supp.zip › dyy032-suppl_data/ije-2017-05-0586-File018.docx]
