# Supplementary material for: The long-term impact of folic acid in pregnancy on offspring DNA methylation: follow-up of the Aberdeen Folic Acid Supplementation Trial (AFAST)
Source: Int J Epidemiol. 2018 Mar 12;47(3):928–37. doi: 10.1093/ije/dyy032 (PMC6005053; doi:10.1093/ije/dyy032)
Supplement: Supplementary Data [file dyy032_supp.zip › dyy032-suppl_data/ije-2017-05-0586-File019.docx]

**S3 Figure** – Differential methylated region plots for the EWAS results in AFAST

a) Folic acid supplement use (low and high dose combined) vs. placebo


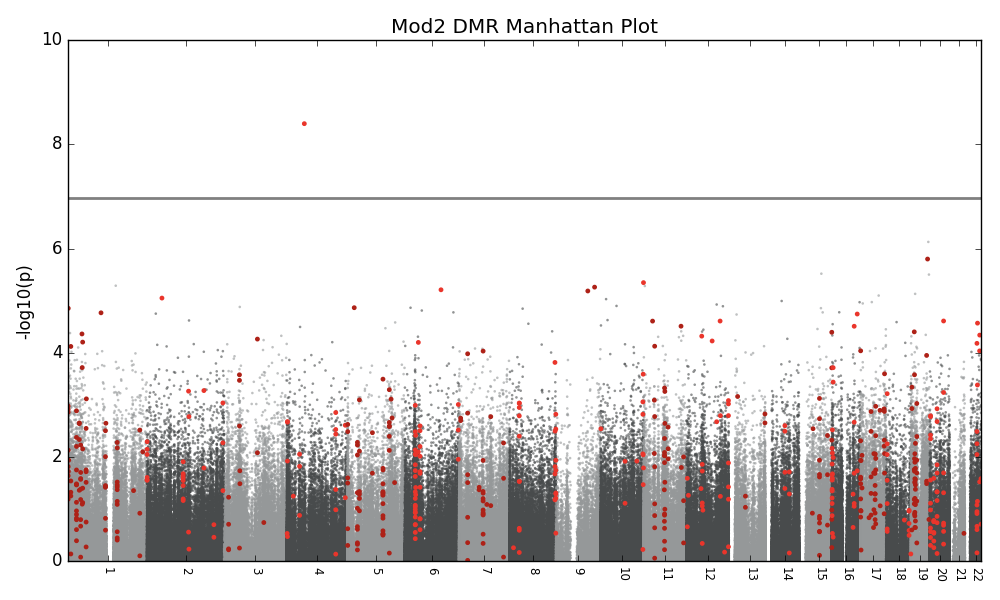


b) High dose vs. placebo


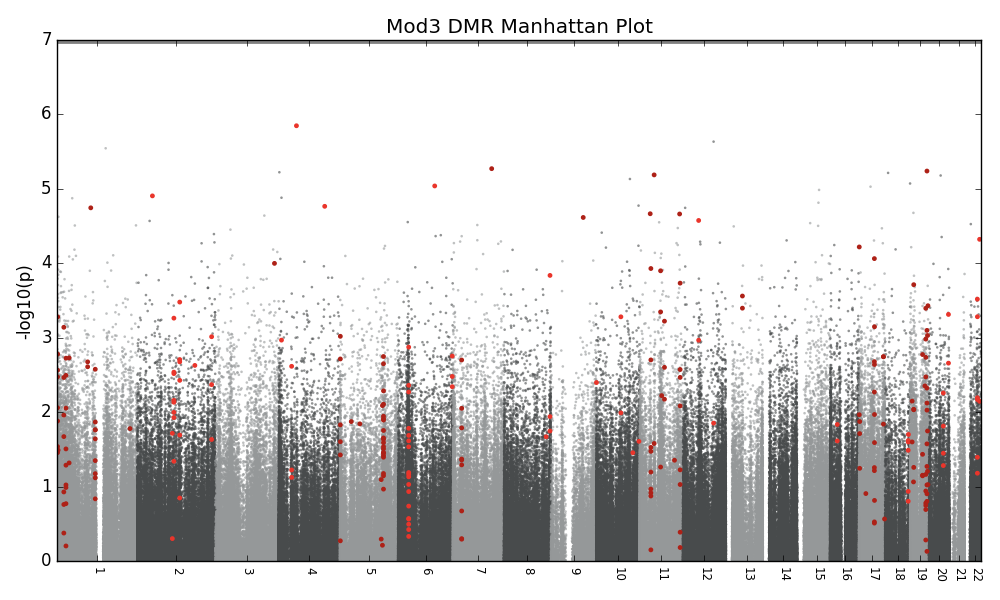


c) Low dose vs. placebo


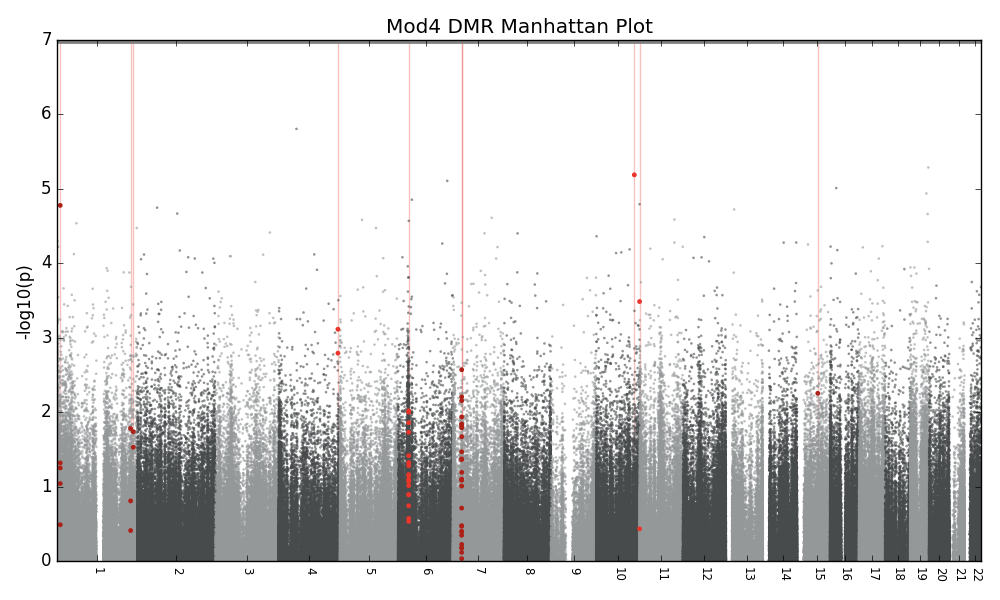


Red dots = individual CpG sites underlying DMRs
