# Supplementary material for: The long-term impact of folic acid in pregnancy on offspring DNA methylation: follow-up of the Aberdeen Folic Acid Supplementation Trial (AFAST)
Source: Int J Epidemiol. 2018 Mar 12;47(3):928–37. doi: 10.1093/ije/dyy032 (PMC6005053; doi:10.1093/ije/dyy032)
Supplement: Supplementary Data [file dyy032_supp.zip › dyy032-suppl_data/ije-2017-05-0586-File020.docx]

**S4 Figure** – Differential methylated region plots for the EWAS of maternal plasma folate*


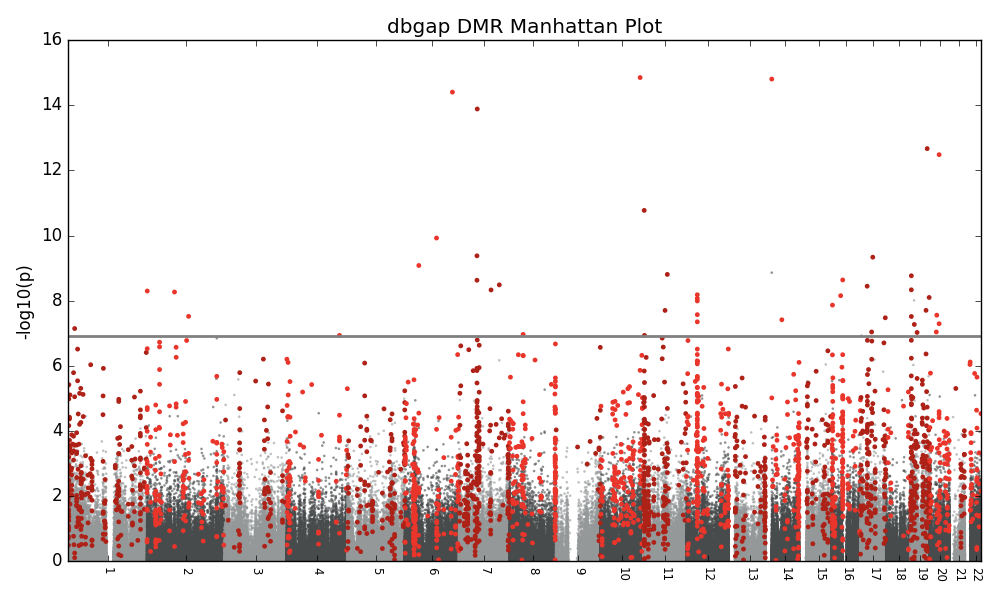


Red dots = individual CpG sites underlying DMRs

*EWAS summary results obtained from dbGAP (phs001059.v1.p1)
